# Appropriate Modeling of Infectious Diseases

**DOI:** 10.1371/journal.pmed.0020239

**Published:** 2005-07-26

When the SARS epidemic showed the first signs of waning, the World Health Organization proclaimed that the turnaround was a testament to the efficient response of health systems worldwide and justified its decisive action in issuing a global alert.

That swift response was partly due to infectious disease experts being able to use models of disease spread, even though SARS was a newly emerging disease, to help plan their next move. In fact, epidemiologists have used mathematical models to predict and understand the dynamics of infectious diseases for more than 200 years. The emergence of diseases such as Ebola, SARS, and West Nile virus, and multi-drug-resistant malaria—as well as the potential for diseases to be introduced by bioterrorism—has attached even greater importance to this management tool.

Models are used to provide information on such infections and predict the effect of alternative courses of action. In this month's *PLoS Medicine*, Helen Wearing and colleagues suggest, however, that many off-the-shelf models are inappropriate for making quantitative predictions because substantial biases have been introduced by two important, yet largely ignored, assumptions. The authors warn that if such biases are not corrected, health authorities risk making overly optimistic health policy decisions.

They begin with the “SEIR” class of models, in which the host population is classified according to infectious status, i.e., individuals are susceptible, exposed, infectious, or recovered. This model assumes that the rate of leaving the exposed or infectious class is constant, irrespective of the time already spent in that class. Although mathematically convenient, this assumption gives rise to exponentially distributed latent (incubation) and infectious periods, which is epidemiologically unrealistic for most infections, say the authors, who suggest instead that it would more sensible to specify the probability of leaving a class as a function of the time spent within the class. Hence, initially, the chance of leaving the class is small but then increases as the mean infectious/incubation period is reached. This assumption would give a more realistic distribution of incubation and infectious periods.[Fig pmed-0020239-g001]

**Figure pmed-0020239-g001:**
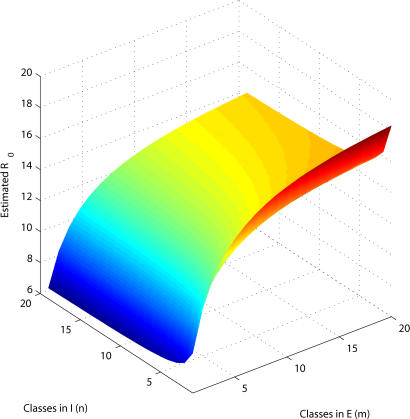
Estimates of variation in the basic reproductive ratio

The authors also note another issue that has received surprisingly little attention in infectious disease models, namely, the influence of incubation and infectious period distributions on the invasion dynamics of an infection into a largely susceptible population—despite its obvious application to emerging infections and possible “deliberate exposure.”

The impact of these differences on models could translate into potentially important public health concerns, say the authors. They tested their theory by using analytical methods to show that, first, ignoring the incubation period or, second, assuming exponentially distributed incubation and infectious periods (when including the incubation period) always resulted in underestimating the basic reproductive ratio of an infection from outbreak data. They then illustrated these points by fitting epidemic models to data from an influenza outbreak. Their results suggested that within a strict management setting, epidemiological details could make a crucial difference.

Although previous studies have shown the importance of using realistic distributions of incubation and infectious periods in endemic disease models, few studies have considered the effects associated with making predictions for an emerging disease. Discrepancies between estimates of reproductive ratio from exponentially distributed and gamma-distributed fits confirm the need to have precise distributions of incubation and infectious periods. Although such data are available from post hoc analyses of epidemics, they are lacking for novel emerging infections. The key point is that uncertainty about these distributions should be incorporated into models when making quantitative predictions.

The take home message is that when developing models for public health use, policy makers need to pay attention to the intrinsic assumptions within classical models. The authors note that while some practitioners are using their approach, most applied epidemiological studies still use models that incorporate exponentially distributed incubation and infectious periods; the authors hope their work will point to the next steps in delivering quantitatively accurate epidemiological models.

